# Risk factors for parental psychopathology: a study in families with children or adolescents with psychopathology

**DOI:** 10.1007/s00787-018-1156-6

**Published:** 2018-04-11

**Authors:** L. W. Wesseldijk, G. C. Dieleman, F. J. A. van Steensel, M. Bartels, J. J. Hudziak, R. J. L. Lindauer, S. M. Bögels, C. M. Middeldorp

**Affiliations:** 1 Department of Biological Psychology, VU University Amsterdam, Van Der Boechorststraat 1, 1081 BT Amsterdam, The Netherlands; 2 Amsterdam Public Health Institute, Amsterdam, The Netherlands; 3 Department of Child and Adolescent Psychiatry/Psychology, Erasmus Medical Center Rotterdam/Sophia Children’s Hospital, Rotterdam, The Netherlands; 4 Research Institute of Child Development and Education, University of Amsterdam, Amsterdam, The Netherlands; 5 UvA Minds, Academic Child and Parent Treatment Center, Amsterdam, The Netherlands; 6 Neuroscience Amsterdam, Amsterdam, The Netherlands; 7 Division of Human Genetics, Department of Psychiatry and Medicine, Center for Children, Youth and Families, University of Vermont, Burlington, USA; 8 Department of Child and Adolescent Psychiatry, Academic Medical Center, Amsterdam, The Netherlands; 9 De Bascule, Academic Center for Child and Adolescent Psychiatry, Amsterdam, The Netherlands; 10 Child Health Research Centre, University of Queensland, Brisbane, Australia; 11 Child and Youth Mental Health Services, Children’s Health Queensland Hospital and Health Service, Brisbane, Australia

**Keywords:** Parental psychopathology, Risk factors, Childhood psychopathology, Parent–offspring associations, Family circumstances

## Abstract

The parents of children with psychopathology are at increased risk for psychiatric symptoms. To investigate which parents are mostly at risk, we assessed in a clinical sample of families with children with psychopathology, whether parental symptom scores can be predicted by offspring psychiatric diagnoses and other child, parent and family characteristics. Parental depressive, anxiety, avoidant personality, attention-deficit/hyperactivity (ADHD), and antisocial personality symptoms were measured with the Adult Self Report in 1805 mothers and 1361 fathers of 1866 children with a psychiatric diagnosis as assessed in a child and adolescent psychiatric outpatient clinic. In a multivariate model, including all parental symptom scores as outcome variables, all offspring psychiatric diagnoses, offspring comorbidity and age, parental age, parental educational attainment, employment, and relationship status were simultaneously tested as predictors. Both 35.7% of mothers and 32.8% of fathers scored (sub)clinical for at least one symptom domain, mainly depressive symptoms, ADHD symptoms or, only in fathers, avoidant personality symptoms. Parental psychiatric symptoms were predicted by unemployment. Parental depressive and ADHD symptoms were further predicted by offspring depression and offspring ADHD, respectively, as well as by not living together with the other parent. Finally, parental avoidant personality symptoms were also predicted by offspring autism spectrum disorders. In families with children referred to child and adolescent psychiatric outpatient clinics, parental symptom scores are associated with adverse circumstances and with similar psychopathology in their offspring. This signifies, without implying causality, that some families are particularly vulnerable, with multiple family members affected and living in adverse circumstances.

**Electronic supplementary material:**

The online version of this article (10.1007/s00787-018-1156-6) contains supplementary material, which is available to authorized users.

## Introduction

Parents whose children suffer from psychiatric symptoms are at risk for psychiatric symptoms themselves. However, it is yet unclear in which families with children assessed at a child and adolescent psychiatric outpatient clinic, parents are most at risk for psychiatric disorders. More insight into these risk factors can facilitate earlier recognition of these families that may benefit from more intensive treatment.

Studies measuring parental symptoms or diagnoses in families with children referred to child and adolescent outpatient clinics repeatedly found increased means or prevalence rates in parents [1–24]. The majority of these studies focused on parental anxiety and depressive symptoms or disorders, but some also showed increased rates of parental attention-deficit/hyperactivity disorder (ADHD) 24 [2, 9, 11, 17] and antisocial personality disorder 24 [1, 2]. In a sample of children referred to a general psychiatric outpatient clinic, overall, 24% of mothers and fathers had symptom scores in the (sub)clinical range on either one of the internalizing or externalizing problem scales 24. These parental symptoms are not always equivalent to their child’s psychiatric problems, e.g., parents can suffer from depressive symptoms while their child has been diagnosed with autism spectrum disorder (ASD), anxiety, ADHD, schizophrenia, oppositional-defiant-, or conduct symptoms [2, 3, 5–11, 13–20, 22, 24].

Several factors may influence the risk for parental psychiatric symptoms, and, these factors may have a different effect for internalizing than externalizing symptoms in parents. There are several reasons why the risk for parental symptoms may depend on the childhood’s disorder. One reason is that the heritability, an important cause of psychiatric disorders to run in families, ranges from 40% (for depression and anxiety) to 80% (for, e.g., ADHD and ASD) [25]. These differences in heritability may result in variation in the risk for psychiatric disorders in parents with children affected by different types of psychopathology. Another reason is that the burden of caring for a child with psychopathology, which may trigger parental psychiatric symptoms, may be different for different childhood psychiatric disorders and may be associated with increased risk for some parental psychiatric disorders but not for others. Results in clinical samples have been mixed. In a study comparing families with children with ASD, ASD + ADHD, or ADHD, depressive scores were found to be highest in the parents of children with ASD, or ASD and ADHD [17]. In a comparison of parents of children with a pure anxiety disorder to parents of children with pure ASD, pure ADHD-combined type, or pure ADHD inattentive type, no differences were found in the level of parental internalizing and externalizing symptoms [18]. The total problem scores were higher in parents of children with the ADHD-inattentive type than in parents of children with an anxiety disorder [18].

Parental psychiatric symptoms may also differ depending on the severity of the offspring symptoms. Parenting stress, which is related to parental psychopathology [17, 18], was observed to be higher when children with ADHD also had co-occurring conduct problems, indicating higher severity [26], but this effect was not seen in families with children with comorbid ADHD and ASD [17]. So overall, comorbidity, which indicates higher severity, seems to be related with an increased parental burden, but results are not conclusive.

General risk factors known to be associated with psychopathology, including financial problems, unemployment, divorce, being a single parent, [27, 28] and demographic characteristics of parent and child, like gender and age, might also influence the likelihood of parental psychiatric symptoms. For example, a large national-claim database study in the United States found the incidence of depression in parents of children diagnosed with autism spectrum disorder to increase with age of the child [15], whereas the age of children with anxiety disorders was not found to influence parental internalizing and externalizing problem scores [18].

No earlier studies have examined the relationship between, on the one hand, family, parent and child characteristics, including child’s psychiatric disorders and the presence of comorbidity, and, on the other hand, a broad range of parental psychiatric symptoms in a clinical sample. This study aims to explore risk factors for parental psychiatric symptoms at the time a child is assessed for psychiatric disorders. We assessed psychiatric symptoms in 1805 mothers and 1361 fathers from 1866 children at the time their child was evaluated in a mental health clinic. The majority of the children were diagnosed with ADHD, autism spectrum disorders, anxiety or depressive disorders. We examined whether family (relationship status), parental (education level, occupational status, age and gender) and offspring characteristics (age, kind of psychiatric diagnosis, and comorbidity) predicted depressive, anxiety, ADHD, avoidant personality, and antisocial personality symptom scores in parents. Knowledge on risk factors help us understand the impact of certain child psychiatric disorders on the parents and which families are particularly vulnerable because both the child and (one of the) parents are affected with psychopathology. Since these characteristics are relatively easily acquired during child evaluations at child and adolescent psychiatric outpatient clinics, they can also provide valuable information on whether additional care for the parents should be considered.

## Methods

### Participants and recruitment

Participants came from four child and adolescent psychiatric outpatient clinics in The Netherlands (de Bascule, GGZ inGeest and UvA Minds in Amsterdam and the Erasmus University Medical Center-Sophia Children’s Hospital (EUMC) in Rotterdam). At the time of the study, children were referred to a child and adolescent psychiatric outpatient clinic mainly by their general practitioner or another health professional. The four clinics offer mental health care to children who have a range of psychiatric problems such as depression, anxiety, autism spectrum disorder, ADHD, and behavioral disorders. The average age of the children (60.4% boys) was 11 years at first referral and of the mothers and fathers 43 and 46 years, respectively (Table 1).

**Table 1:** Descriptives of offspring and parental characteristics. Mean age (SD) and DSM diagnoses for the children (%) are displayed at the top parental mean (SD) age, education level (%), employment status (%), relationship status (%) and number of parents (%) with a score in the (sub)clinical range are displayed at the bottom

	Boys (N = 1127)	Girls (N = 739)
Mean age (SD)	10.80 (3.12)	12.00 (3.59)
DSM diagnosis (*n* (%) )		
ADHD	586 (52%)	224 (30.3%)
ASD	262 (23.2%)	83 (11.2%)
Disruptive behavior	61 (5.4%)	44 (6%)
Depression	54 (4.8%)	76 (10.3%)
Anxiety	192 (17%)	239 (32.3%)
Trauma	45 (4%)	52 (7%)
Tic	13 (1.2%)	5 (.7%)
Eating disorders	4 (.4%)	37 (5%)
NOS	67 (5.9%)	45 (6.1%)
Other	78 (6.9%)	71 (9.6%)
More than one diagnoses	242 (21.5%)	143 (19.2%)

	Mothers (*N* = 1805)	Fathers *(N* = 1361)
Mean age (SD)	43.50 (6.22)	46.22 (6.47)
Education level (*n* (%) )		
Low	262 (15.1%)	219 (16.9%)
Intermediate	475 (27.3%	344 (26.6%)
High	1000 (57.6%)	730 (56.5%)
Employment status		
Yes	1407 (78.6%)	1219 (90.6%)
No	384 (21.4%)	127 (9.4%)
Relationship status		
Yes	1201 (67.9%)	1072 (78.8%)
No	568 (32.1%)	289 (21.2%)
(Sub)clinical range total (*n* (%) )	643 (35.7%)	451 (32.8%)
Per analyzed domain:		
Depressive	263 (14.6%)	176 (12.8%)
Anxiety	129 (7.2%)	83 (6.0%)
Avoidant	130 (7.2%)	156 (11.3%)
ADHD	232 (12.9%)	156 (11.3%)
Antisocial	125 (6.9%)	103 (7.5%)

Employment status: having a job yes/no. Relationship status: together with biological parent yes/no (where ‘no’ includes single parenthood from birth onwards or being divorced later on)

*ADHD* attention-deficit/hyperactivity disorders, *ASD* autism spectrum disorders, *NOS* disorders of infancy, childhood, or adolescence not otherwise specified

Data were collected between April 2010 and December 2016. In all clinics, the parents of the child were asked to rate their child’s problems as part of the first assessment. If possible, both parents were asked to complete the questionnaires. Only parents who did not have a sufficient knowledge of the Dutch language were excluded from participation. All studies were approved by the Central Ethics Committees of the participating institutions. For the current study, families were selected if the parental survey was filled in by the biological parent. We excluded data of children who did not fulfill the criteria of a psychiatric diagnosis after assessment (*n* = 30). In total, data were analyzed for 1805 mothers (96.73%) and 1361 fathers (72.94%) from 1866 unrelated children.

### Measures

*Demographic information* regarding the child’s age, the parent’s age, parent’s education level, employment, and relationship status was collected from a questionnaire that was administered before the first visit. Parental education level was defined in three categories: low (primary school, lower vocational schooling and lower secondary schooling), middle (intermediate vocational schooling and intermediate/higher secondary schooling) and high (higher vocational schooling, university and post graduate). Parents were employed or unemployed (yes/no). Relationship status was coded as being together with other biological parent yes/no and ‘no’ includes single parenthood from birth onwards or being divorced later on.

*Parental psychiatric symptoms* were measured with the Adult Self Report (ASR), which is part of the Achenbach System of Empirically Based Assessment (ASEBA). For more information on the ASR, we refer to the website (aseba.org) and the manual [29] where the specific items and (sub)clinical threshold scores can be found. In the ASR, adults rate 120 items on a three-point scale (0 = not true, 1 = somewhat true, 2 = very true). The ASR offers, besides the commonly used empirical scales, DSM-oriented scales that are associated with the presence or absence of DSM diagnoses [30, 31]. We analyzed the following DSM-oriented scales: depressive symptoms, anxiety symptoms, avoidant personality symptoms, ADHD symptoms, and antisocial personality symptoms.

DSM diagnoses in children were assessed by a multi-disciplinary team of clinicians based on the information obtained from the parents and child in diagnostic interviews and in the questionnaires collected before the first assessments combined with the teacher reports on the child’s psychiatric problems and sometimes observations in the classroom. The diagnoses were categorized following the DSM–IV diagnostic categories [32]: attention-deficit/hyperactivity disorders (ADHD), autism spectrum disorders (ASD), disruptive behavior disorders, depressive disorders, anxiety disorders, post-traumatic stress disorder, tic disorders, eating disorders and, disorder of infancy, childhood, or adolescence not otherwise specified (NOS). Adjustment disorder with mixed anxiety and depressed mood was added to depressive disorders. Adjustment disorder with disturbance of conduct was added to disruptive behavior disorders. This left 151 children with a diagnosis that could not be categorized (e.g., selective mutism or somatization disorder), who were listed as “other”. A binary measure of comorbidity was constructed based on whether the child received one or more DSM diagnoses.

### Analyses

As dichotomizing the parental scores into a normal and (sub)clinical score results in a loss of information on the variation and thereby in a loss of statistical power [33], continuous sum scores of symptoms were analyzed. To get a first impression of the associations between the family, child, and parent characteristics, and the parental psychiatric scores, means and standard deviations for maternal and paternal psychiatric symptom scores per psychiatric problem scales were obtained as a function of childhood diagnoses, comorbidity within the child, parental education level, parental employment status, and parental relationship status.

**Fig. 1:**
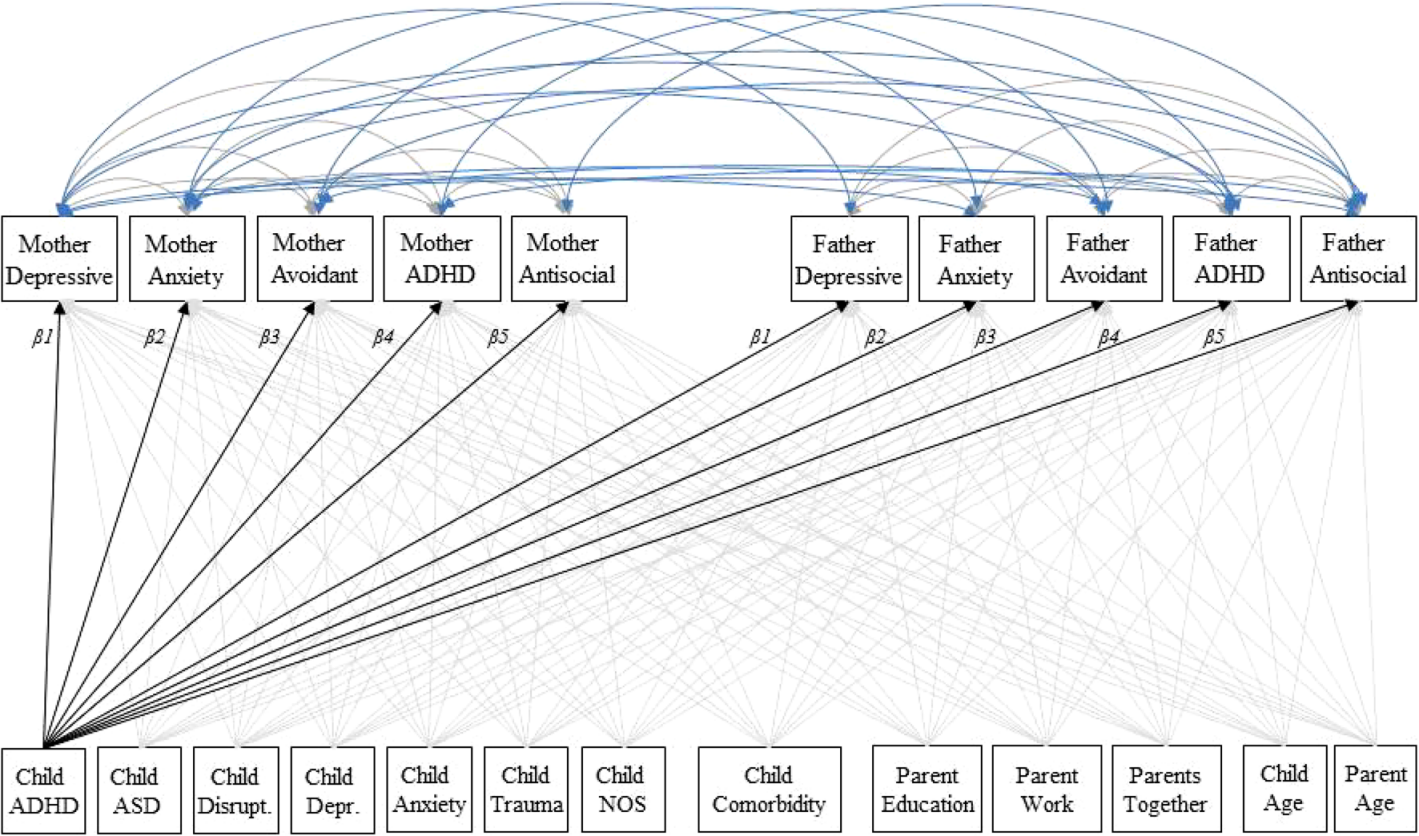
The multivariate model: the psychiatric symptom scores are correlated within the parent and between mothers and fathers. The child’s diagnoses, comorbidity and the demographic variables (i.e., education level, employment and relationship status and age of the child and parent) predict the parental psychiatric symptom scales. The betas were constrained to be equal for mothers and fathers (β1–β5). ADHD: Attention-deficit/hyperactivity disorders. *ASD* autism spectrum disorders, *Disrupt* disruptive behavior disorders, *Depr* depressive disorders, *NOS* disorders of infancy, childhood, or adolescence not otherwise specified

Next, we performed a multivariate multiple regression analysis in Mplus, in which all maternal and paternal psychiatric symptom scores were predicted by all child’s psychiatric diagnostic categories (i.e., depression, ADHD), comorbidity, the age of the child, the age of the parent, the education level of the parent, employment of the parent, and the relationship status of the biological parents. In the model, we allowed the parental symptom scores to correlate within the parent and between mothers and fathers [34] (see Fig. 1). Since the thirteen predictors were correlated, we used the software ‘matSpD’ to calculate that a *p* value of < 0.004 as a threshold for statistical significance for the regression coefficients is appropriate to correct for multiple testing [35, 36].

We did not differentiate between childhood psychiatric disorders in boys or girls, due to our sample size (e.g., paternal psychiatric symptom ratings were only available for 23 girls with disruptive behavior disorder). A visual inspection of the means of the parental psychiatric symptom scores by child’s diagnosis, comorbidity and family characteristics for boys and girls separately showed no consistent gender differences.

## Results

### Descriptive statistics

Characteristics of the parents and children are shown in Table 1 for the total sample. Supplementary Table 1 shows the characteristics for each of the four clinics. In the children, boys mainly received diagnoses of ADHD (52%), ASD (23.2%), or anxiety disorder (17%), while girls were mostly diagnosed with anxiety disorder (32.3%), ADHD (30%), ASD (11.2%), or a depressive disorder (10.3%). Since the numbers of children with tic and eating disorders were low, these categories were not included as predictors in the analyses. The group of “other” diagnoses was not included as a predictor either, due to the variety among the diagnoses. In the parents, 35.7% of the mothers and 32.8% of the fathers scored in the (sub)clinical range on at least one of the psychiatric symptom domains at time of the first assessment of their child. The highest percentages of parents scoring above the threshold were for depressive symptoms, ADHD symptoms and, in fathers only, avoidant personality symptoms with percentages varying between 11 and 15%. As a comparison, for each scale, the threshold for (sub)clinical symptoms is set at the 93rd percentile, based on assessments in the general population (see for further details the ASEBA manual for the adult forms), i.e., in the general population 7% score above the (sub)clinical threshold for each scale.

### Predictions

**Fig. 2:**
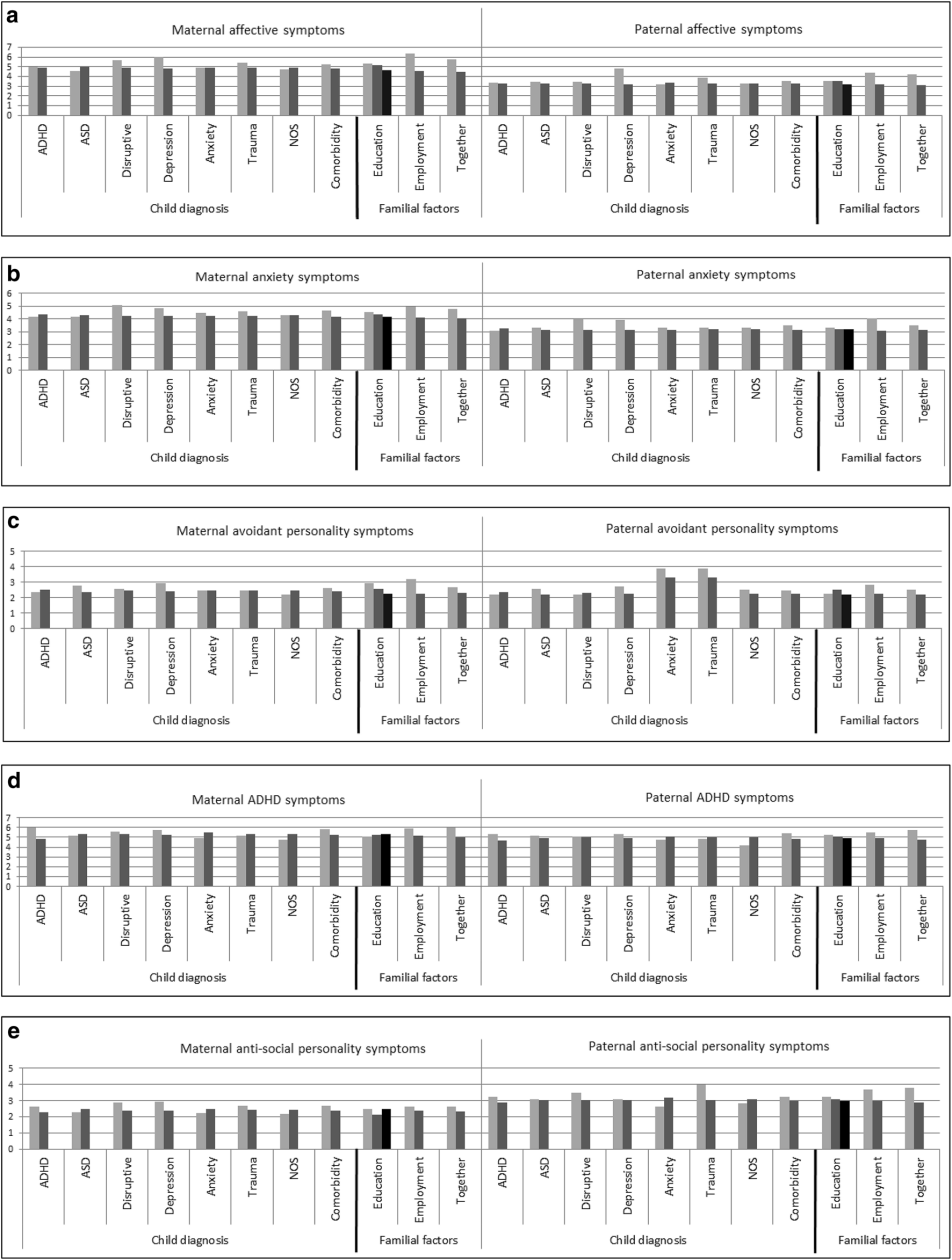
Means of the parental affective (**a**), anxiety (**b**), avoidant personality (**c**), ADHD (**d**) and anti-social personality problem (**e**) scores by child’s diagnosis (ADHD, ASD, disruptive, depression, anxiety, trauma or NOS versus all other diagnoses) and by comorbidity in the child (yes/no) education level (low, middle, high), work status (no/yes) and whether the biological parents of the child are together (no/yes). *ADHD* attention-deficit/hyperactivity disorders, *ASD* autism spectrum disorders, *Disruptive* disruptive behavior disorders, *NOS* disorders of infancy, childhood, or adolescence not otherwise specified

Figure 2a–e depict the mean maternal and paternal scores on the five different psychiatric symptom scales by child’s diagnosis versus all other diagnoses and by education level of the parent, employment, relationship status, and the child’s comorbidity. The effects of the predictors were similar in direction and magnitude in mothers and fathers (see Fig. 2a–e). Therefore, in the multivariate model, the betas were constrained to be equal for mothers and fathers (see Fig. 1).

Table 2 shows the regression coefficients derived from the multivariate model. It can be seen that parents who are unemployed have increased scores for depressive symptoms, anxiety symptoms, avoidant personality symptoms and ADHD, with a particularly large effect for depressive symptoms (regression coefficient 1.36 while the other coefficients were around 0.8). Parents who are not together with the biological parent have increased scores for depressive symptoms and ADHD (coefficients both around 1). Age of the child or parent did not significantly predict the risk for any of the parental psychiatric symptoms, nor did parental education level. Some childhood diagnoses predicted specific parental psychiatric symptoms. Offspring ADHD predicted parental ADHD symptoms, offspring depression predicted parental depressive symptoms, and offspring ASD predicted parental avoidant personality symptoms. The prediction was strongest for depressive symptoms (coefficient 1.65) and lowest for avoidant personality symptoms (coefficient 0.63). Anti-social personality symptoms were not predicted by any offspring diagnosis and there were not any significant predictions by offspring trauma, disorder of infancy, childhood, or adolescence NOS or child comorbidity.

**Table 2:** Standardized regression coefficients for the multivariate multi-group analysis with the parental psychiatric symptom scales predicted by the child diagnoses, comorbidity (yes/no), age of the child and parent, education level of the parent (low–middle–high) and relationship (yes/no together) and employment status (yes/no employed)

	Depressive		Anxiety		Avoidant		ADHD		Ant-social	
	*β*	SE	*β*	SE	*β*	SE	*β*	SE	*β*	SE
Child diagnosis										
ADHD	0.76	0.30	0.19	0.22	0.07	0.18	1.17*	0.34	0.15	0.20
ASD	0.60	0.30	0.34	0.22	0.63*	0.20	0.54	0.34	− 0.05	0.20
Disruptive behavior	0.61	0.47	0.73	0.30	0.05	0.32	0.24	0.53	0.29	0.36
Depression	1.65*	0.43	0.58	0.28	0.37	0.25	0.76	0.49	0.24	0.32
Anxiety	0.38	0.29	0.37	0.28	0.15	0.19	0.09	0.33	− 0.37	0.20
Trauma	0.81	0.54	0.27	0.37	0.21	0.33	0.05	0.52	0.13	0.32
NOS	0.60	0.41	0.44	0.30	0.17	0.25	− 0.05	0.44	− 0.19	0.28
Comorbidity	− 0.52	0.29	0.02	0.21	− 0.07	0.19	− 0.07	0.34	0.21	0.21
Characteristics										
Child age	0.02	0.03	0.05	0.02	0.01	0.02	0.02	0.03	− 0.01	0.02
Parent age	0.01	0.02	− 0.01	0.01	0.00	0.01	− 0.01	0.02	0.03	0.01
Education level	− 0.14	0.12	0.04	0.08	− 0.15	0.07	0.08	0.12	0.03	0.07
Employment	− 1.36*	0.29	− 0.82*	0.19	− 0.80*	0.17	− 0.87*	0.28	− 0.28	0.16
Relationship	− 0.96*	0.23	− 0.37	0.14	− 0.16	0.13	− 1.02*	0.24	-0.46	0.18

*ADHD* attention-deficit/hyperactivity disorders, *ASD* autism spectrum disorders, *NOS* disorders of infancy, childhood, or adolescence not otherwise specified

*p < 0.004

## Discussion

This is, to our knowledge, the first study to examine which factors predict a broad range of psychiatric symptoms scores in mothers and fathers of families with children assessed for psychopathology in an outpatient psychiatric clinic. Prevalence rates of parental scores in the (sub)clinical range were 35.7% for mothers and 32.8% for fathers for one of the psychiatric symptom scales, mainly depressive symptoms, ADHD symptoms or, in fathers, avoidant personality symptoms. The multivariate analyses showed the importance of family risk factors (namely employment and relationship status) for parental psychopathology, and of child’s diagnoses of ADHD, depression and autism spectrum disorders, specifically for parental ADHD, depressive symptoms and avoidant personality symptoms, respectively. Our findings indicate that a part of the families with children assessed for a psychiatric disorder are particularly vulnerable since they live in adverse family circumstances and multiple family members suffer from psychopathology.

The high prevalence rates for parental psychopathology found in this clinical population with the largest proportion of participating fathers so far (72.94% of the children), confirms again the necessity of parental screening of both mothers and fathers when a child is referred to a child and adolescent psychiatric outpatient clinic [24, 34]. Our findings on the associations of parental symptom scores with family circumstances (employment and relationship status) were also in line with previous research [27, 28], indicating how problems can accumulate in families. Furthermore, we found parental symptom scores to be predicted by similar psychopathology in their child, regarding depression, ADHD and parental avoidant personality symptoms. Contrary to the study by van Steijn et al. [17], offspring autism spectrum disorders did not predict depressive symptoms in parents. However, avoidant personality symptoms, that we found to be predicted by offspring ASD, were not included in the former study and are also associated with depressive symptoms, with correlations around 0.6 [34]. Future studies should clarify whether ASD in offspring is indeed mainly related to avoidant personality problems instead of depression.

In contrast to an earlier study on parents of children with ASD [15], but in line with study on parents of children with anxiety disorders [18], we did not find an effect of the age of the child on parental psychopathology. It could be that the age effect is confined to parents of children with ASD as parents may become more aware of the continuing disabilities, or the problems may become more debilitating on school and social functioning when their child becomes older, and may hinder them to become independent adults. In the current analysis, this effect would then be diluted by the lack of the effect of the child’s age for other disorders (e.g., anxiety disorders).

We performed the analyses on the continuous symptom scores. As a sensitivity analysis, we additionally performed univariate logistic regression analyses including all variables from the multivariate analyses (the seven child psychiatric diagnoses, comorbidity, child age and gender, parent age and gender, parental relationship status and occupational status) as predictors for parental (sub)clinical scores yes/no. In these analyses, only the prediction of a parental score in the (sub)clinical range for ADHD by the offspring ADHD diagnosis showed a significant effect (see Supplementary Table 2), in addition to the significant effects of unemployment and not being together. The absence of significant effects of offspring depression and ASD on parental depressive and avoidant symptoms, respectively, could well be due to the loss of statistical power in analysing dichotomous variables.

This study has several strengths, such as the large sample size overall and, in particular the inclusion of a large group of fathers, the broad assessment of psychiatric symptoms in the parents, the inclusion of children with various psychiatric diagnoses, and the use of a statistical method that takes into account the associations between psychiatric symptoms. There are also several limitations. Although response rates were fairly high (at least 60%, after the pilot study), not all parents reported on their psychiatric symptoms. The percentage of employed mothers and fathers in our sample was higher than in the general Dutch population (mothers: 67.9 vs. 61.6%, fathers: 90.6 vs. 71%), while the percentage of parents not being together (32.1% in case of mothers participation, 21.2% for fathers) was lower than the 39.6% divorce rate in the Netherlands (of which 55.8% are divorces between partners with at least one child versus 44.2% without a child) [37–39]. This may suggest a response bias in our sample, which has most likely led to an underestimation of the prevalence rates for parental psychopathology. Psychiatric diagnoses in the children were mostly based on clinician’s views which were checked in multidisciplinary intake teams and not on standardized interviews, although, if indicated, diagnostic instruments to assess ASD, such as the Autism Diagnostic Observation Schedule (ADOS) and the Autism Diagnostic Interview (ADI) were used [40, 41]. On the other hand, results based on a naturalistic design such as in the current study, accurately reflect current clinical practice. Finally, we did not have information on the duration of adverse circumstances, such as unemployment or being a single parent family. It could be that longer duration is associated with lower risk due to adaptations by the parents. This is a subject for future study.

We would like to emphasize that our findings do not imply causal effects. The association between unemployment and parental psychiatric symptoms could evenly be due to the severity of the child’s problems which resulted in the parent both having to quit the job and suffering from psychopathology. Transmission of genetic risk goes from parents to children. Still, parent and offspring psychopathology are mutually associated. For example, remission of maternal depression has a positive effect on their children’s psychiatric symptoms [42] and treatment of a child’s psychopathology has a positive effect on mother’s depressive symptoms [43]. A longitudinal study in a population-based sample showed that the child’s mental health status at 5 and at 14 years independently predicted maternal mental health 21 years post birth of the child, while adjusting for environmental risk factors and mother’s prenatal mental health [44]. Future experimental and longitudinal studies can provide more insight into the direction of effect.

Our results do indicate which families with children with psychopathology are at a higher risk of having affected parents. Providing additional care and treatment to these parents may be beneficial for the whole family. Overall, we now know that parents experience higher rates of psychiatric symptoms and that holds for both mothers and fathers. The associations between parent and offspring psychopathology have also been shown to be similar in magnitude for mothers and fathers [24]. Furthermore, previous analyses in part of this sample showed that parents of a child with psychopathology are more alike regarding their psychiatric symptoms than parents in the general population, i.e., if one of the parents suffers from psychiatric symptoms the other parent has an increased chance of psychopathology [34]. We now add that the circumstances of a family, i.e., employment and relationship status, are associated with increased risk for parental psychopathology in addition to specific predictions by offspring psychopathology. Future research should investigate how the mothers, fathers, and children in these multiple affected families, can be helped most effectively. For example, is it more effective to treat the psychopathology of the child to reduce the psychopathology of the parent, or to treat the psychopathology of the parent to reduce the psychopathology of the child, or is it more effective to treat the psychopathology of the family member affected him/herself? This again argues for bridging the gap between child psychiatry and adult psychiatry and establish clinics that are able to provide integrated care for the whole family.

## Electronic supplementary material

Below is the link to the electronic supplementary material.

Supplementary material 1 (DOCX 18 kb)
