# Supplementary material for: Risk factors for parental psychopathology: a study in families with children or adolescents with psychopathology
Source: Eur Child Adolesc Psychiatry. 2018 Apr 11;27(12):1575–84. doi: 10.1007/s00787-018-1156-6 (PMC6245117; doi:10.1007/s00787-018-1156-6)
Supplement: Supplementary file 1 — Supplementary material 1 (DOCX 18 kb) [file 787_2018_1156_MOESM1_ESM.docx]

**Supplementary material**

Table 1: *Descriptives of the sample per child and adolescent psychiatry outpatient clinic. Mean (SD) age, education level (%), employment status (%), relationship status (%) and mean (SD) symptom scores for mothers and fathers per psychiatric symptom scale.*

|  | **Bascule (N=183)** | **GGZ inGeest (N=410)** | **UvA Minds (N=919)** | **Rotterdam (N=354)** |
| --- | --- | --- | --- | --- |
| **Age (Mean (SD))**  Children  Mothers  Fathers | 10.83 (3.82)  42.42 (6.48)  45.92 (6.67) | 11.70 (3.77)  42.92 (7.04)  47.10 (6.68) | 11.55 (3.03)  45.04 (5.46)  47.34 (5.98) | 10.33 (3.24)  40.35 (5.68)  42.76 (6.20) |
| **Education level (n (%))**  Mothers  *Low*  *Intermediate*  *High* | 38 (22.6%)  54 (32.1%)  76 (45.2%) | 56 (15%)  107 (28.7%)  210 (56.3%) | 71 (8%)  191 (21.5%)  628 (70.6%) | 101 (31.6%)  130 (40.6%)  89 (27.8%) |
| Fathers  *Low*  *Intermediate*  *High* | 24 (21.8%)  31 (28.2%)  55 (50%) | 35 (15.2%)  68 (29.4%)  128 (55.4%) | 83 (12%)  155 (22.4%)  455 (65.7%) | 104 (32.7%)  110 (34.6%)  104 (32.7%) |
| **Employment status (n (%))**  Mothers employed  Fathers employed | 117 (69.6%)  96 (85%) | 280 (72.7%)  209 (87.4%) | 749 (81.9%)  659 (91.5%) | 275 (81.6%)  305 (92.4%) |
| **Relationship status (n (%))**  Biological parents together | 123 (69.1%) | 227 (58.4%) | 620 (67.5%) | 264 (77.9%) |
| **Symptom scores (Mean (SD))**  Mothers  *Depression*  *Anxiety*  *Avoidant*  *Attention deficit/hyperactivity*  *Antisocial*  Fathers  *Depression*  *Anxiety*  *Avoidant*  *Attention deficit/hyperactivity*  *Antisocial* | 5.28 (4.72)  4.66 (3.07)  2.48 (2.40)  5.04 (4.03)  2.39 (2.36)  3.44 (4.36)  3.28 (2.64)  2.40 (2.66)  4.86 (4.73)  3.30 (3.99) | 5.27 (4.94)  4.37 (3.07)  2.50 (2.63)  5.12 (4.29)  2.50 (2.55)  3.01 (3.13)  2.98 (2.18)  1.95 (2.28)  4.28 (4.19)  3.06 (3.07) | 4.78 (4.02)  4.18 (2.63)  2.24 (2.24)  5.56 (4.46)  2.52 (2.62)  3.32 (3.36)  3.14 (2.41)  2.33 (2.40)  5.30 (4.14)  3.23 (2.88) | 4.66 (4.23)  4.17 (2.62)  2.87 (2.48)  4.99 (4.08)  2.08 (1.99)  3.46 (3.48)  3.42 (2.51)  2.35 (2.41)  4.73 (3.76)  2.47 (2.51) |

Table 2: *The percentage of parents with a score in the (sub)clinical range per psychiatric symptom scale (italic) by the child diagnosis (bold) / all other child diagnoses .*

|  | **ADHD** | **ASD** | **Behavioral** | **Depression** | **Anxiety** | **Trauma** | **NOS** |  |
| --- | --- | --- | --- | --- | --- | --- | --- | --- |
|  | Yes / No | Yes / No | Yes / No | Yes / No | Yes / No | Yes / No | Yes / No |  |
| *Depressive* | 14.3% / 13.4% | 13.6% / 13.9% | 16% / 13.7% | 20.8% / 13.3% | 13.3% / 14% | 22.2% / 13.4% | 10.2% / 14% |  |
| *Anxiety* | 6.5% / 6.9% | 6.8% / 6.6% | 8% / 6.6% | 12.3% / 6.3% | 6.8% / 6.6% | 11.1% / 6.5% | 5.1% / 6.8% |  |
| *Avoidant* | 8.9% / 9.1% | 11.1% / 8.5% | 7.4% / 9.1% | 11.3% / 8.8% | 9.3% / 8.9% | 9.8% / 9% | 6.6% / 9.1% |  |
| *ADHD* | 15.2% / 9.8%* | 12.7% / 12.1% | 9.3% / 12.4% | 17.5% / 11.8% | 10.7% / 12.7% | 12.2% / 12.4% | 7.7% / 12.5% |  |
| *Antisocial* | 9% / 5.7% | 6.1% / 7.4% | 10.5% / 7% | 9.4% / 7% | 4.7% / 8% | 10.5% / 7% | 6.1% / 7.2% |  |

**p* < 0.004. ADHD: Attention deficit/hyperactivity disorders. ASD: Autism spectrum disorders. NOS: Disorders of infancy, childhood, or adolescence Not Otherwise Specified.
